# Deaths Associated with Pediatric Hepatitis of Unknown Etiology, United States, October 2021–June 2023

**DOI:** 10.3201/eid3004.231140

**Published:** 2024-04

**Authors:** Olivia Almendares, Julia M. Baker, David E. Sugerman, Umesh D. Parashar, Sarah Reagan-Steiner, Hannah L. Kirking, Paul A. Gastañaduy, Jacqueline E. Tate, Hepatitis of Unknown Etiology Group

**Affiliations:** Centers for Disease Control and Prevention, Atlanta, Georgia, USA

**Keywords:** hepatitis, child, viruses, adenovirus infections, acute disease, fatal outcome, United States

## Abstract

During October 2021–June 2023, a total of 392 cases of acute hepatitis of unknown etiology in children in the United States were reported to Centers for Disease Control and Prevention as part of national surveillance. We describe demographic and clinical characteristics, including potential involvement of adenovirus in development of acute hepatitis, of 8 fatally ill children who met reporting criteria. The children had diverse courses of illness. Two children were immunocompromised when initially brought for care. Four children tested positive for adenovirus in multiple specimen types, including 2 for whom typing was completed. One adenovirus-positive child had no known underlying conditions, supporting a potential relationship between adenovirus and acute hepatitis in previously healthy children. Our findings emphasize the importance of continued investigation to determine the mechanism of liver injury and appropriate treatment. Testing for adenovirus in similar cases could elucidate the role of the virus.

During October–November 2021, clinicians at a single children’s hospital in Alabama, USA, identified 5 previously healthy children with acute hepatitis of unknown etiology who tested positive for adenovirus species F, type 41 (*1*); that pathogen was not previously considered a common cause of severe acute hepatitis in immunocompetent persons. In subsequent months, similar cases were identified internationally (*2*), prompting the launch of a nationwide public health investigation into pediatric acute hepatitis of unknown etiology in the United States. On April 21, 2022, the Centers for Disease Control and Prevention (CDC) issued a health advisory recommending that clinicians consider adenovirus testing in pediatric patients with hepatitis of unknown etiology and that patients meeting the following criteria be reported to jurisdictional health departments: <10 years of age with hepatitis manifested by elevated (>500 U/L) aspartate aminotransferase (AST) or alanine aminotransferase (ALT) and onset since October 1, 2021 (*3*).

As of June 6, 2023, health departments reported to CDC a total of 392 children meeting the hepatitis of unknown etiology case criteria. Worldwide, as of July 8, 2022, a total of 1,010 probable cases were reported to the World Health Organization on the basis of a closely aligned case criterion: children <16 years experiencing acute, non-‒A-E hepatitis with AST or ALT >500 IU/L since October 1, 2021. Despite similar case definitions, the United States was one of the few countries to report fatalities (*2*).

Severe acute hepatitis is known to have several potential etiologies, including drug-induced, genetic, metabolic, infectious, or immune-mediated causes (*4*). Adenovirus is a well-known cause of severe hepatitis in immunocompromised persons; it often has a direct pathologic effect on the liver (*4*–*7*). Conversely, few reported cases describe a potential association between adenovirus and severe acute hepatitis in immunocompetent children; the mechanism of liver injury in immunocompetent children is poorly understood (*8*–*11*). Previous studies estimate that 30%–50% of acute liver failure occurring in children without prior liver disease was of undetermined cause (*7*,*12*). However, adenovirus was commonly detected among children tested for the virus in recent investigations in the United States (116/275, 42%) (*13*) and United Kingdom (*14*) (170/258, 66%), raising the question of whether adenovirus may be an underrecognized cause of pediatric acute hepatitis.

We describe the characteristics of children who died after hepatitis of unknown etiology and were reported to CDC as part of the national-level investigation. Second, we describe the potential involvement of adenovirus and other pathogens in the development of severe acute hepatitis among these children. Last, we contextualize the deaths within the broader national-level investigation, describing similarities and differences between this subset of patients with the most severe outcomes and the overall patient population.

## Methods

### Study Population and Data Collection

This study includes all deaths reported to CDC among US children with acute hepatitis of unknown etiology with onset of October 1, 2021‒June 6, 2023. A child was reported if they initially met the criteria defined in the CDC health advisory and if the child’s death was related to their hepatitis episode, regardless of the time between hepatitis onset and death. Reported cases underwent further investigation including medical chart abstractions, caregiver interviews, laboratory testing, and, when possible, pathologic evaluation of liver tissue specimens. Health departments or clinicians completed medical chart abstractions using a standardized case report form to collect demographics, medical and illness history, laboratory test results, treatment received, and outcomes. Interviews were conducted with a parent or caregiver at the discretion of the health department to obtain additional information on each patient’s symptoms; healthcare use; ill contacts at home, school, or daycare; and other potential exposures such as diet and travel. In addition, autopsy reports, discharge summaries, death certificates, and other available medical records were requested for further review to better understand the course of illness and elucidate the possible role of adenovirus or other pathogens in development of severe acute hepatitis and death.

### Specimen Collection and Testing

Laboratory testing for clinical management was not standardized; testing varied by individual patient and by clinical judgment. Most respiratory and fecal specimens were tested locally by multiplex PCR panel. In addition, testing for adenovirus by nucleic acid amplification testing (e.g., PCR or quantitative PCR) of blood, respiratory, or stool samples was requested at the discretion of the treating clinician and conducted at a clinical or reference laboratory. Available residual adenovirus-positive blood, stool, respiratory, and tissue specimens were sent to select reference laboratories or CDC for adenovirus typing using Sanger sequencing of the 6 hypervariable regions of the hexon gene (*15*). Formalin-fixed, paraffin-embedded (FFPE) tissues from liver biopsy, explant, or autopsy underwent routine evaluation at the clinical institutions or medical examiner’s office; available specimens were submitted to CDC. CDC’s evaluation of specimens included histopathology, immunohistochemistry (IHC), and conventional PCR and sequencing for adenovirus, as well as IHC and PCR for other infectious etiologies, as indicated based on the pathologic findings and clinical and epidemiologic history.

### Record Review

All deaths reported to CDC as part of the hepatitis of unknown etiology investigation through June 6, 2023, are noted in this report. Some children were excluded as cases by the reporting jurisdiction as additional clinical information became available and an etiology was identified. For the remaining children, clinical and epidemiology teams at CDC reviewed all available medical records and related laboratory and pathological evaluations to develop consensus opinions on possible etiologies and confirm each child met the reporting criteria. Two pediatricians (P.A.G. and H.L.K.) independently evaluated reported underlying medical conditions to determine whether those conditions may have contributed to development of or increased risk for acute hepatitis. We excluded children with an etiology for their hepatitis from further investigation and analysis. We describe here in detail the children with no known or suspected etiology after review of all available records.

### Statistical Analysis

We used descriptive statistics to summarize the data and results. We analyzed all data in R version 4.2.1 (The R Foundation for Statistical Computing, https://www.r-project.org). CDC reviewed the investigation protocol and confirmed that it was conducted consistent with applicable federal law and CDC policy (45 C.F.R. part 46.102(l)(2), 21 C.F.R. part 56; 42 U.S.C. §241(d); 5 U.S.C. §552a; 44 U.S.C. §3501 et seq.).

## Results

### Eligibility and Record Availability

During the national investigation, a total of 23 pediatric deaths with hepatitis onset occurring during October 1, 2021–June 6, 2023, were reported to CDC from 13 US jurisdictions: Florida, Georgia, Indiana, Michigan, Missouri, Nebraska, New York City, Pennsylvania, Puerto Rico, South Carolina, Tennessee, Texas, and Wisconsin (Table 1). Fourteen (60.9%) of the 23 children did not meet the reporting criteria defined in the health advisory on the basis of detailed medical record and laboratory testing review by the reporting jurisdiction or CDC. The most common reason for exclusion was identification of an alternative etiology for the hepatitis (n = 12). Alternative etiologies were complications of sepsis from pathogens other than adenovirus (n = 6), congenital conditions (n = 3), and unintentional injury (n = 3) (Table 1). After review of all information provided to CDC, 8 (34.8%) children were confirmed to meet the reporting criteria and are further described in this report. One child’s eligibility could not be determined because records were unavailable.

**Table 1:** Eligibility and exclusions among fatally ill children reported to the pediatric acute hepatitis of unknown etiology investigation, United States, October 1, 2021–June 6, 2023

Description	No. (%)
Total reported	23 (100.0)
Meets reporting criteria*	8 (34.8)
Does not meet reporting criteria	14 (60.9)
Unknown	1 (4.3)
Reason for exclusion	
Age ≥10 y	1 (7.1)
Onset date before October 1, 2021	1 (7.1)
Alternative etiology†	12 (85.7)
Complications of sepsis, pathogens other than adenovirus	6 (50.0)
Congenital conditions	3 (25.0)
Unintentional injury‡	3 (25.0)

*Child <10 y of age with elevated (>500 U/L) aspartate aminotransferase or alanine aminotransferase and hepatitis of unknown etiology with onset since October 1, 2021. †Some children presented with complex clinical courses. The cause of hepatitis was not conclusive, but an alternative etiology or etiologies were considered likely. Categorizations were based upon available records. ‡Unintentional injuries include suffocation and acetaminophen toxicity.

Record availability differed by child (Table 2). A case report form was completed for all children, whereas an exposure questionnaire was available for 5 (62.5%). An autopsy had been conducted for 4 children; all those reports were available for review. A discharge summary was available for all 8 children and a death certificate was available for 6. Other available records included emergency department visit notes, admission history and physical notes, progress and consultation notes, and additional laboratory and radiologic testing results. FFPE autopsy tissue specimens from 3 children were submitted to CDC for evaluation; of those 3 children, 1 also had native liver explant submitted, and 1 a liver biopsy.

**Table 2:** Record availability for 8 children with hepatitis of unknown etiology, United States, October 1, 2021–June 6, 2023*

Patient ID	Case report form	Exposure questionnaire	Autopsy conducted	Autopsy report	Discharge summary	Death certificate/ cause of death	Other records	Pathology specimen(s) evaluated at CDC†
1	Y	N	N	NA	Y	Y	N	N
2	Y	N	Y	Y	Y	N	Y	Y
3	Y	Y	N	NA	Y	Y	N	N
4	Y	Y	Unknown	N	Y	Y	N	N
5	Y	Y	Y	Y	Y	Y	N	Y
6	Y	Y	Y	Y	Y	Y	N	N
7	Y	Y	Y	Y	Y	Y	Y	Y
8	Y	N	N	NA	Y	N	Y	N
Total no. (%)	8 (100.0)	5 (62.5)	4 (50.0)	4 (50.0)	8 (100.0)	6 (75.0)	3 (37.5)	3 (37.5)

*Y indicates the record was available to CDC; N indicates the record was not available to CDC. CDC, Centers for Disease Control and Prevention; NA, not applicable. †Available pathology specimens were sent to CDC for evaluation and further characterization. Tissue analyses conducted at the treating facility are not included here.

### Demographics and Clinical Characteristics at Initial Visit

The median age at acute hepatitis onset was 2.4 (range 0.2–6.3) years (Table 3). Five patients (62.5%) were male and 3 (37.5%) female; 5 (5/8, 62.5%) identified as Hispanic or Latino ethnicity. At initial visit, 2 children were immunocompromised and receiving immunosuppressive therapy (1 for ongoing cancer treatment and 1 after liver transplant), 2 other children had conditions that may have increased their risk of developing hepatitis or complications of adenovirus infection in the right clinical circumstance (1 was small for gestational age; 1 had history of poor weight gain and possible gastrointestinal disorder), and 4 children had no known underlying conditions (Table 3; Appendix Table 1). Children with underlying conditions that may have increased their risk and those with no known underlying conditions are henceforth called previously healthy.

**Table 3:** Clinical and demographic characteristics of 8 children with hepatitis of unknown etiology, United States, October 1, 2021–June 6, 2023 *

Characteristic	Value
Demographic characteristics	
Age	
Median age (range)	2.4 (0.2–6.3)
0–11 mo	2 (25.0)
1–2 y	4 (50.0)
3–9 y	2 (25.0)
Sex	
F	3 (37.5)
M	5 (62.5)
Race/ethnicity	
Hispanic or Latino	5 (62.5)
Black, non-Hispanic	2 (25.0)
White, non-Hispanic	1 (12.5)
Clinical characteristics	
Underlying conditions at initial visit	
Immunocompromised†	2 (25.0)
Comorbidities that may have increased risk for hepatitis‡	2 (25.0)
No underlying conditions	4 (50.0)
Reported signs and symptoms§	
Respiratory	6/8 (75.0)
Gastrointestinal	7/8 (87.5)
Hepatic	8/8 (100.0)
Systemic	7/8 (87.5)
Laboratory findings	
Highest alanine transaminase, median (range), U/L	4,852 (219–5,648)
Highest aspartate aminotransferase, median (range), U/L	7,500 (786–10,000)
Highest total bilirubin, median (range), mg/dL	13.7 (1.3–19.4)
Highest international normalized ratio, median (range)	8.0 (1.4–13.0)
Highest ammonia, median (range), µmol/L	126.0 (60.3–801.0)
Duration of symptoms before admission, d	
Median (range)	7 (1–36)
0–7	5 (62.5)
8–14	1 (12.5)
>15	2 (25.0)
Duration of hospital stay, d	
Median (range)	14.5 (<1– 18)
0–2	2 (25.0)
3–14	2 (25.0)
>15	4 (50.0)
Physical findings	
Hepatomegaly	5/8 (62.5)
Hepatic encephalopathy	4/8 (50.0)
Splenomegaly	2/7 (28.6)
Ascites	3/7 (42.9)
Outcomes¶	
Acute liver failure	7 (87.5)
Liver transplant	2 (25.0)

*Values are no. (%) except as indicated. †Immunocompromised at the time of initial presentation. One child immunosuppressed due to ongoing cancer treatment. One child immunocompromised due to a liver transplant. ‡One child was small for gestational age and 1 child had a history of poor weight gain and possible gastrointestinal disorder. §Symptoms are not mutually exclusive; all children reported multiple symptoms. Respiratory signs/symptoms include cough, congestion, runny nose, shortness of breath, conjunctivitis, sore throat, or wheezing. Gastrointestinal signs/symptoms include diarrhea, nausea, vomiting, or abdominal pain. Hepatitis signs/symptoms include dark colored urine, pale stool, jaundice, or scleral icterus. Systemic signs/symptoms include fatigue, decreased appetite, or fever. See Appendix Table 2 for data by patient. ¶Not mutually exclusive. Acute liver failure was defined based on documentation written in the medical chart or medical chart abstraction indicating acute liver failure occurred.

All children manifested hepatitis signs and symptoms (i.e., dark colored urine, pale stool, jaundice, or scleral icterus); most also reported other systemic symptoms (87.5%), gastrointestinal symptoms (87.5%), and respiratory symptoms (75.0%) during their illness course (Table 3; Appendix Table 2). The median duration of symptoms before emergency department or inpatient admission was 7 (range 1–36) days (Figure). Laboratory evaluations showed elevated transaminases; median peak ALT was 4,852 (range 219–5,648) U/L and AST 7,500 (range 786–10,000) U/L. Median peak total bilirubin level was 13.7 (range 1.3–19.4) mg/dL and INR was 8.0 (range 1.4–13.0) (Table 3). Before the hepatitis episode, all but 1 child had received >2 doses of hepatitis B vaccine >3 weeks before their hepatitis episode (62.5% had 3 or 4 doses) and 5 children had received 1 or 2 doses of hepatitis A vaccine >3 months before their hepatitis episode. One child had received COVID-19 vaccine >3 months before the episode.

**Figure:**
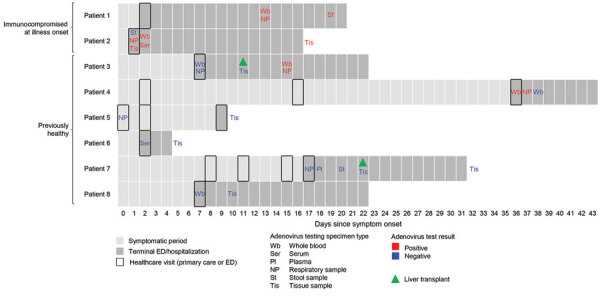
Health status at initial visit and timeline of clinical course for 8 children fatally ill with hepatitis of unknown etiology, United States, October 1, 2021–June 6, 2023. Symptom onset refers to the first symptom reported for the child including respiratory, gastrointestinal, hepatic, or systemic symptoms. Patients 1 and 2 were immunocompromised at the time of initial visit to emergency department or inpatient admission. Patient 3, initially immunocompetent, tested positive only after receiving a liver transplant during initial admission; before the transplant, the child had tested negative for adenovirus in respiratory, whole blood, and liver specimens. ED, emergency department.

### Adenovirus and Other Pathogen Testing

For every child, >2 specimen types were tested for adenovirus by multiplex PCR panel (using respiratory or stool specimens) or by quantitative PCR (using blood specimens); the most common specimen types tested were blood (n = 7), respiratory (nasopharyngeal, n = 6), FFPE liver tissue (n = 6), and stool/rectal swab (n = 3) (Table 4). Four of the 8 children tested positive for adenovirus; each was positive in both respiratory specimens and whole blood. One child also tested positive in stool and another in both serum and liver tissue. Blood specimens had the highest adenovirus detection rate (4/7, 57.1%), particularly whole blood (4/5, 80%). Adenovirus typing data were available for 2 children.

**Table 4:** Individual patient adenovirus testing for children with hepatitis of unknown etiology, United States, October 1, 2021–June 6, 2023*

Patient ID	Result and day of testing								
	Any specimen	Respiratory†	Stool‡	Blood (any§)	Whole blood	Serum	Plasma	FFPE tissue tested at clinical institution¶	FFPE tissue tested at CDC#
Patient 1**	P, day 13	P (B7), day 13	P (B7), day 19	P (NT), day 13	P (NT), day 13				
Patient 2**	P, day 1	P (NT), day 1	N†† day 1	P (NT), day 2	P (C1), day 2	P (NT), day 2		P (NT), day 1	P (C), day 1
Patient 3	P, day 15	P‡‡ (NT), day 15		P‡‡ (NT), day 15	P‡‡ (NT), day 15			N, day 11	
Patient 4	P, day 36	P (NT), day 37		P (NT), day 36	P (NT), day 36				
Patient 5	N, day 0	N, day 0							N, postmortem
Patient 6	N, day 2			N, day 2		N, day 2		N, postmortem	
Patient 7	N, day 17	N, day 17	N, day 20	N, day 18			N, day 18		N, day 22
Patient 8	N, day 7			N, day 7	N, day 7			N, day 10	
Total no. positive/no. tested (%)	4/8 (50.0%)	4/6 (66.7%)	1/3 (33.3%)	4/7 (57.1%)	4/5 (80.0%)	1/2 (50.0%)	0/1 (0.0%)	1/4 (25.0%)	1/3 (33.0%)

*Blank cells indicate not tested. P, positive; N, negative; NT, not typed; B7, adenovirus species B, type 7; C1, adenovirus species C, type 1; C, adenovirus species C; FFPE, formalin-fixed, paraffin-embedded. †Respiratory specimens were tested by multiplex PCR panel. Specific panel type used may have varied based on manufacturer used by the diagnostic or reference laboratory. ‡Stool specimens were tested by nucleic acid amplification testing (e.g., PCR). §Includes any blood specimen type (whole blood, serum, plasma). Blood specimens were tested by real-time PCR or quantitative RT-PCR. ¶Specimens were tested for adenovirus by immunohistochemistry (IHC) at the clinical institution. Tissue from Patient 2 additionally underwent testing by PCR at the clinical institution. #Specimens were tested for adenovirus by IHC and PCR at CDC. **Child considered to be immunocompromised at the time of hepatitis onset. ††Stool was tested by PCR for adenovirus type 40/41 and was negative. ‡‡Adenovirus test was negative before patient’s liver transplant and positive after transplant.

Of the 4 children who tested positive for adenovirus, 2 (patients 1 and 2) had immunocompromising conditions before hepatitis onset; 1 was positive for adenovirus species B, type 7 (respiratory and stool samples), and 1 was positive for species C, type 1, in whole blood and species C in liver tissue. The third child (patient 3), initially immunocompetent, tested positive for adenovirus (type unknown) after becoming immunocompromised after a liver transplant received during the initial hospital stay; before the liver transplant, the child had tested negative for adenovirus in respiratory, whole blood, and liver specimens. The fourth child (patient 4) who tested positive for adenovirus (type unknown) had no underlying conditions but was <1 year of age. Of the 4 children who were negative for adenovirus (patients 5–8), all were considered previously healthy (Table 4; Figure; Appendix Table 1).

All children tested positive for >1 viral or bacterial pathogen (Table 5). After adenovirus, the most commonly detected pathogens were bacterial pathogens isolated in culture from normally sterile sites (blood or joint fluid, n = 4), followed by *Clostridium difficile* (stool specimens, n = 2), rhinovirus/enterovirus (respiratory specimens, n = 2), and respiratory syncytial virus (respiratory specimens, n = 2). In addition, 1 child had serologic evidence of acute Epstein-Barr virus infection. Of note, none of the children had evidence of acute SARS-CoV-2 infection; 2 children were reported to have had previous SARS-CoV-2 infection on the basis of serologic results in their medical records.

**Table 5:** Positive pathogen detections in children with hepatitis of unknown etiology, United States, October 1, 2021–June 6, 2023*

Patient ID	Respiratory specimen	Stool specimen	Normally sterile site (blood or joint)†	Total no. pathogens detected
1	Adenovirus	Adenovirus	Adenovirus; *Streptococcus pneumoniae*	2
2	Adenovirus; respiratory syncytial virus		Adenovirus	2
3	Adenovirus; rhinovirus/enterovirus		Adenovirus	2
4	Adenovirus; rhinovirus/enterovirus		Adenovirus	2
5			*Staphylococcus hominis*; *Corynebacterium*; *Moraxella* non-liquefaciens‡	3
6	Respiratory syncytial virus		Epstein-Barr virus	2
7		*Clostridioides difficile*		1
8		*Clostridioides difficile*		1

*Table includes all pathogens for which a positive result was detected; it does not include other testing performed for which there were no positive results (e.g., respiratory and/or gastrointestinal panels, other viral testing). Other infections may have been present, but testing was not performed or results were inconclusive. Most respiratory and GI specimens were tested as part of a multiplex PCR panel, but panels used by clinical laboratory varied. All testing was performed at the discretion of treating clinicians. † Bacterial pathogens were isolated on culture from a normally sterile site such as blood or joint fluid. Testing for viral pathogens was conducted using real-time PCR or quantitative real-time PCR testing, or by serologic assay to detect recent viral antibodies (e.g., IgM). ‡*Staphylococcus hominis*, Corynebacterium, and *Moraxella* non-liquefaciens may reflect contamination of specimen collection and not necessarily infection.

Liver specimens from 6 children underwent routine pathological examination at the clinical institution or medical examiner’s office; 3 children had FFPE liver tissue specimens submitted to CDC. In 3 children (patients 3, 6, and 8), FFPE liver tissue specimens were negative for adenovirus by IHC at the clinical institution. Of the 3 children for whom FFPE liver tissue specimens were submitted to CDC, 2 (patients 5 and 7) were negative for adenovirus by IHC and PCR at CDC. FFPE liver tissue from 1 child (patient 2) was positive by IHC and PCR at the clinical institution and CDC, and adenovirus species C was detected by PCR at CDC. In addition, adenovirus species C was detected in whole blood and respiratory samples (Table 4). In the same child (patient 2), liver tissue pathology was consistent with classic adenovirus hepatitis including smudge cells. Pathologic findings seen in liver varied in the other 5 children (e.g., acute hepatitis, massive necrosis, interface hepatitis).

### Outcomes

The median duration of hospitalization was 14 days (range <1–18 days). Seven (87.5%) children had acute liver failure as noted on the medical abstraction form; 2 of those underwent liver transplantation during acute hospitalization (Table 3; Figure). Seven children had a death certificate or autopsy report available that described the cause of death, including liver-related causes (viral hepatitis, acute cholestatic hepatitis, acute liver failure, hepatic encephalopathy, acute liver transplant rejection), cardiopulmonary causes (acute respiratory distress syndrome, cardiopulmonary arrest), cerebral herniation, or multiorgan failure. Among the 4 children who tested positive for adenovirus, 3 had adenovirus noted in the death certificate or autopsy report. Adenovirus was not mentioned on the death certificate for the fourth adenovirus-positive child (patient 3), who tested positive only after receiving a liver transplant.

## Discussion

The deaths reported during the national investigation of pediatric acute hepatitis of unknown etiology represent a group of young children with diverse medical histories and courses of illness, likely reflecting the medical and etiologic complexity of pediatric hepatitis and the broad case criteria used for the US investigation. Multiple children tested positive for adenovirus and other pathogens. Adenovirus typing data was available for 2 adenovirus-positive children. Of the 4 children who tested positive for adenovirus, most had underlying medical conditions at the time of adenovirus detection that likely put them at greater risk for severe infection and death. The death of 1 previously healthy child with adenoviremia demonstrates the continued importance of adenovirus testing and investigating the potential association between adenovirus and hepatitis in healthy children.

The United States is one of the few countries to report fatalities associated with pediatric acute hepatitis of unknown etiology (*2*), which may reflect minor differences in the case definitions implemented by countries (*16*,*17*), variation in application of those definitions for case finding, or the large size of the US population, enabling identification of rare outcomes. Of note, more than half of the initially reported fatalities in young children with hepatitis were later determined not to meet the case definition, most commonly because an etiology for their hepatitis was ultimately identified.

The diversity and complexities of illness among the children precluded identification of many commonalities; however, we were able to group the children into 3 broad categories. The first category describes a rare but recognized relationship between adenovirus infection and hepatitis: severe hepatitis or acute liver failure in an immunocompromised patient. Three of the 4 children who tested positive for adenovirus fall into this category because they were immunocompromised from immunosuppressive treatment or immunodeficiency at the time of adenovirus detection. The second category of children, of which 4 children were identified in this investigation, encompasses those who were previously healthy and tested negative for adenovirus. Hepatitis among those children may have a wide range of possible etiologies. As previously mentioned, pediatric acute liver failure can have several potential etiologies (*4*); earlier studies estimate that 30%–50% may be of undetermined cause (*7*,*12*). The findings among the fatally ill children reported here and previous studies on pediatric acute liver failure underscore the importance of standardized and comprehensive testing to identify underlying etiologies in children with indeterminate acute liver failure.

The third category encompasses the 1 previously healthy child in this study whose hepatitis appeared to be related to adenovirus, as evidenced by adenoviremia. Although age (<1 year) may be considered a potential risk factor, the child had no known underlying medical conditions or immunodeficiency that would predispose to severe illness or complications from adenovirus infection. Detection of rhinovirus/enterovirus in a respiratory sample was the only other notable laboratory finding. The child had neither an acute nor prior history of SARS-CoV-2 infection. No liver tissue was available for analysis; the relationship between adenovirus infection and liver injury, if any, remains unclear. However, the presence of adenoviremia in conjunction with hepatitis of unknown etiology in this child bears similarity to the original cluster of children in Alabama (*1*) and to other case reports described elsewhere (*18*). The findings from this child and previous literature (*8*–*11*) emphasize the importance of considering adenovirus in the differential diagnosis for pediatric acute hepatitis, despite uncertainty regarding the pathologic mechanism.

The children described in this article are a unique subset of the overall patient population identified in the investigation of acute hepatitis of unknown etiology. Similar to members of the overall patient population, the fatally ill children illustrate a diverse range of possible hepatitis etiologies. The fatally ill children were more likely to have underlying conditions and differed in adenovirus testing results (*13*). Underlying conditions were rare (6%) in the overall patient population, whereas 2 of the 8 fatally ill children had significant underlying medical conditions that increased their risk for hepatitis. Several adenoviruses were detected among the overall patient population, including species B (type 7), C (types 1, 2, 6), and F (types 40, 41). Although adenovirus type 41 is a main focus in the national-level investigation and the most commonly detected type among the nonfatal cases (20/30, 67%), it was not detected in the 2 adenovirus-positive fatal cases that had typing results. Rather, those children were infected with adenovirus types typically seen in severe illness (species B, type 7) or upper respiratory illness (species C, type 1) and reported to be associated with hepatitis in immunocompromised patients (*19*,*20*). Typing was not available for the remaining 2 adenovirus-positive children; however, adenovirus was detected both in respiratory (nasopharyngeal swabs) and whole-blood specimens, suggesting possible infection with an adenovirus other than type 41. Adenovirus 41, which is typically associated with gastrointestinal infections (*20*,*21*), is rarely identified in respiratory specimens (*22*). Last, a high proportion of children in the overall patient population and the fatalities are Hispanic/Latino. Investigation into whether Hispanic/Latino children are at higher risk for diseases and death is needed.

The first limitation of this investigation is that we attempted to construct the clinical context of each child’s illness via a secondary analysis of available medical and laboratory data, but we did not have full access to comprehensive medical records. Limited record availability, complexity and heterogeneity in illness course, and differences in clinical evaluations (e.g., laboratory testing) complicated summarization and full comparison of hepatitis cases. Although we detected multiple pathogens among the children, determining their contribution to hepatitis or death was not possible. Similarly, interpreting the role of adenovirus in the clinical course of illness was constrained by variations in specimen type, timing, and frequency of adenovirus testing. Reports from a related, previously published investigation in Alabama (*1*) suggested differences in the sensitivity of detection by specimen type (highest sensitivity in whole blood) that could add further uncertainty to the interpretation of adenovirus testing results. However, all children were tested for the virus in multiple specimen types, often including blood. Although evaluation was not available at the time of this report, investigation is warranted into the potential role of adeno-associated virus type 2, a parvovirus hypothesized to have either a causal relationship or lead to more severe liver disease in co-infection with adenovirus (*23*,*24*). Last, we could not assess multiple potentially relevant host-related factors that may have affected the risk of illness, clinical course, or treatment, among them genetic or autoimmune predisposition (*24*), socioeconomic status, or behavioral factors (e.g., healthcare-seeking behavior).

The deaths reported during the US investigation of pediatric hepatitis of unknown etiology likely represent a diverse range of etiologies in both the cause of hepatitis and cause of death. This report describes 3 children who demonstrated a recognized relationship between adenovirus and acute hepatitis in immunocompromised persons; it also contributes to the growing evidence suggestive of a potential relationship between adenovirus and acute hepatitis in previously healthy children and the importance of testing for adenovirus early in the course of illness. Further investigation of adeno-associated virus type 2, adenovirus, and other potential helper viruses (e.g., human herpesvirus 6 and 7) (*23*) will contribute to understanding the mechanism of liver injury, if any, and the role of any cofactors or co-infections, particularly among a diverse population of children with no known underlying conditions. If adenovirus or other viral pathogens are contributing to the development of hepatitis, discovery of this relationship may enable clinicians to identify the cause of hepatitis in some future cases in which the etiology and appropriate treatment would have otherwise been unknown.

Appendix Additional information about deaths associated with pediatric hepatitis of unknown etiology, United States, October 2021–June 2023.
