# Supplementary material for: Deaths Associated with Pediatric Hepatitis of Unknown Etiology, United States, October 2021–June 2023
Source: Emerg Infect Dis. 2024 Apr;30(4):644–53. doi: 10.3201/eid3004.231140 (PMC10977843; doi:10.3201/eid3004.231140)
Supplement: Appendix — Additional information about deaths associated with pediatric hepatitis of unknown etiology, United States, October 2021–June 2023. [file 23-1140-Techapp-s1.pdf]

Article DOI: https://doi.org/10.3201/eid3004.231140

EID cannot ensure accessibility for supplementary materials supplied by authors. Readers who have difficulty accessing supplementary content should contact the authors for assistance.

Deaths Associated with Pediatric Hepatitis of Unknown Etiology, United States, October 2021–June 2023

Appendix

Results

Appendix Table 1. Individual patient demographics and clinical data for 8 fatally ill children with hepatitis of unknown etiology, October 1, 2021–June 6, 2023, United States*

Patient ID	Age group, y	Underlying condition(s) (at time of initial presentation) that may predispose the child to hepatitis	Initial presenting location	AST levels upon ED admission	ALT levels upon ED admission
Patient 1	≥3	Yes†	ED	1,719	300
Patient 2	1–2	Yes†	ED	44	42
Patient 3	≥3	No‡	PCP	6,186	4,359
Patient 4	<1	No	PCP	9,619	4,852
Patient 5	<1	Maybe§	ED	756	202
Patient 6	1–2	Maybe§	ED	Unknown	5,000
Patient 7	1–2	No‡	PCP	148	492
Patient 8	1–2	No	PCP	8,415	5,035

*ED, emergency department; PCP, primary care provider.
†Both children with underlying conditions were immunocompromised, one was undergoing cancer treatment and one had a prior liver transplant.
‡ Received liver transplant post hepatitis onset.
§Underlying conditions that, in the right clinical circumstance, may have increased the child's risk of developing hepatitis or complications of adenovirus infection. One child was small for gestational age. The other child had a history of poor weight gain and possible gastrointestinal disorder.

Appendix Table 2. Individual patient data including signs and symptoms for 8 fatally ill children with hepatitis of unknown etiology from October 1, 2021–June 6, 2023, United States*

Signs and symptoms	Patient 1	Patient 2	Patient 3	Patient 4	Patient 5	Patient 6	Patient 7	Patient 8	Total n/N (%)
Duration of symptoms before final ED/hospital admission (d)	2	1	7	36	9	2	17	7	
Respiratory signs/symptoms									
Cough/congestion/runny nose	X			X	X	X		X	5/8 (62.5)
Shortness of breath		X							1/8 (12.5)
Conjunctivitis (pink eye)					X				1/8 (12.5)
Sore throat									0/8 (0.0)
Wheezing									0/8 (0.0)
Gastrointestinal signs/symptoms									
Diarrhea		X	X	X	X		X	X	6/8 (75.0)
Nausea/vomiting			X	X	X	X	X		5/8 (62.5)
Abdominal pain		X	X		X	X	X	X	6/8 (75.0)
Hepatic signs/symptoms									
Dark-colored urine			X		X		X		3/8 (37.5)
Pale stool					X		X	X	3/8 (37.5)
Jaundice/scleral icterus	X	X	X	X	X	X	X	X	8/8 (100.0)
Systemic signs/symptoms									
Fatigue		X	X	X	X	X	X		6/8 (75.0)
Decreased appetite		X	X	X	X	X	X		6/8 (75.0)
Fever	X	X		X	X				4/8 (50.0)

*"X" indicates the sign or symptom was present; blank indicates the sign or symptom was not present.
